# Supplementary figures and images for: Glucocorticoid Receptor-Regulated Enhancers Play a Central Role in the Gene Regulatory Networks Underlying Drug Addiction
Source: Front Neurosci. 2022 May 16;16:858427. doi: 10.3389/fnins.2022.858427 (PMC9149415; doi:10.3389/fnins.2022.858427)

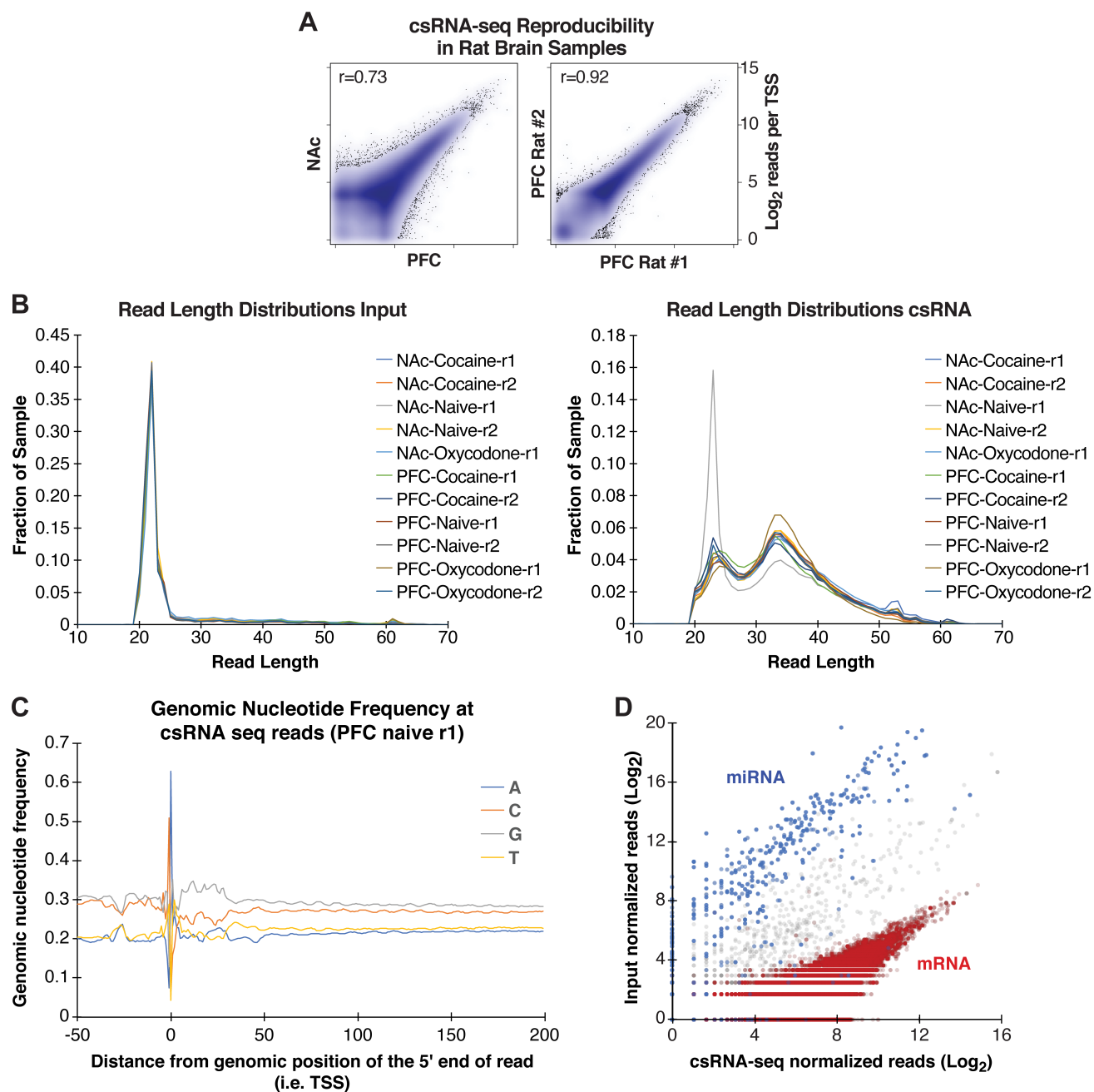

Supplement: Supplementary Figure 1 — csRNA-seq data in rat brain tissues. (A) Variation in csRNA-seq levels at each Transcriptional Start Site Region (TSR) between tissues (NAc vs. PFC) or between replicates (PFC r1 vs. r2) in samples from naive rat brains. (B) Read length distribution for input libraries (left) and csRNA-seq libraries (right). Input libraries show a strong spike at 21 nt corresponding to mature miRNA. (C) Nucleotide frequencies at csRNA-seq reads shown for the PFC naive-r1 library. (D) Read counts at the annotated promoters (5′ end of transcripts −/+ 200bp) with blue dots indicating miRNA transcripts, red dots mRNA transcripts, and grey dots other transcripts (snRNAs, snoRNAs, etc.). csRNA-seq and input data corresponding to the PFC naive-r1 sample is shown. [file Image_1.pdf]

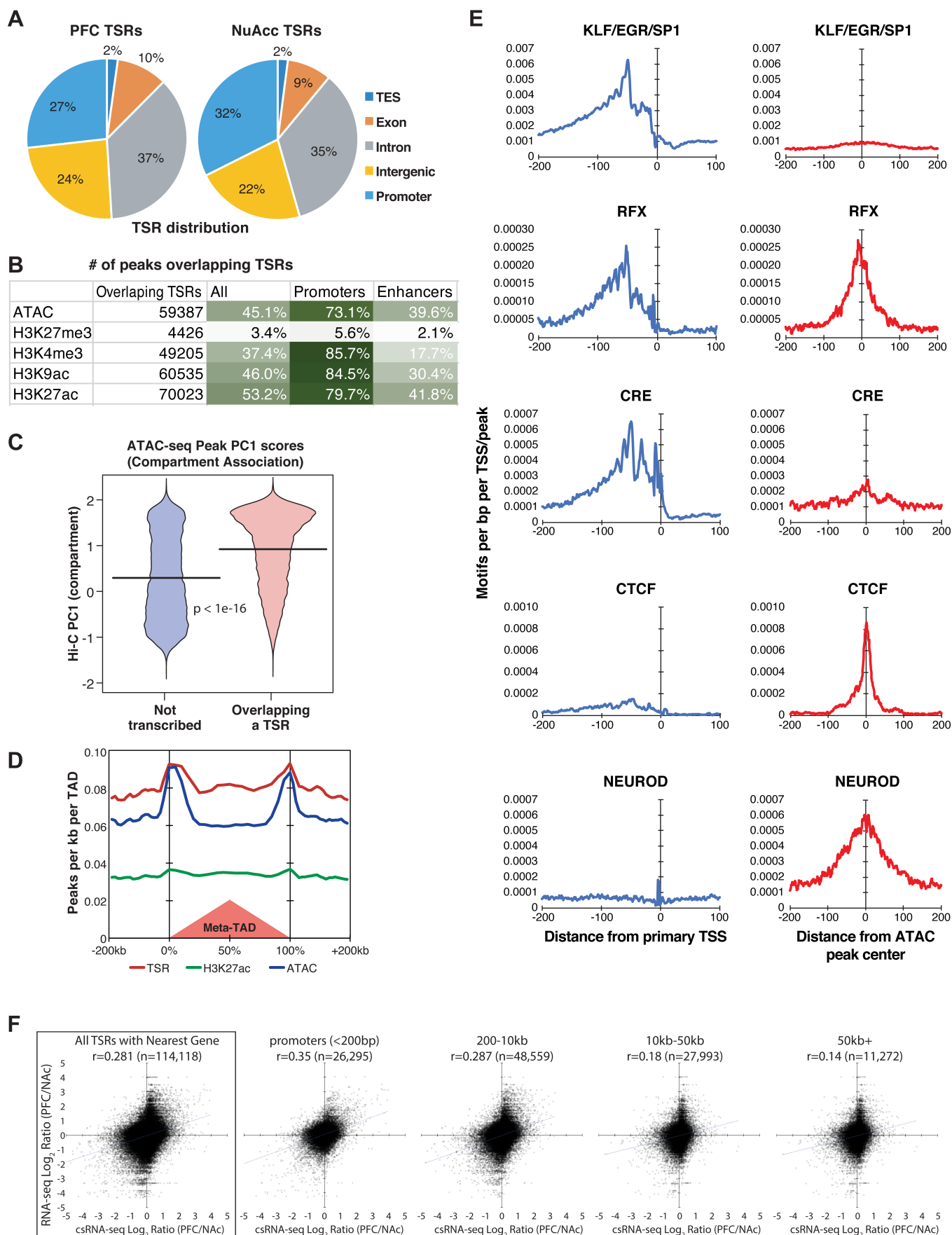

Supplement: Supplementary Figure 2 — Identification and characterization of Transcriptional Start Sites (TSRs) from csRNA-seq data. (A) Pie charts showing the distribution of TSRs in different genomic regions. (B) Fraction (%) of promoter- and enhancer-associated TSRs that overlap peaks identified from ATAC-seq or ChIP-seq for several histone marks. The total numbers of overlapping peaks are reported. (C) Violin plot showing the distribution of Hi-C PC1 values for ATAC-seq peaks that are not transcribed or overlapping a TSR. (D) Histogram showing the distribution of TSRs, H3K27Ac and ATAC-seq peaks around TAD regions identified by Hi-C. (E) Location of several TFs motifs with respect to the primary TSS from csRNA-seq TSRs and the center of ATAC peaks genome-wide. (F) Scatter plots of transcript level differences (Log2 ratio) between NAc and PFC in RNA-seq vs csRNA-seq for TSRs located at different distances from gene TSS. [file Image_2.pdf]

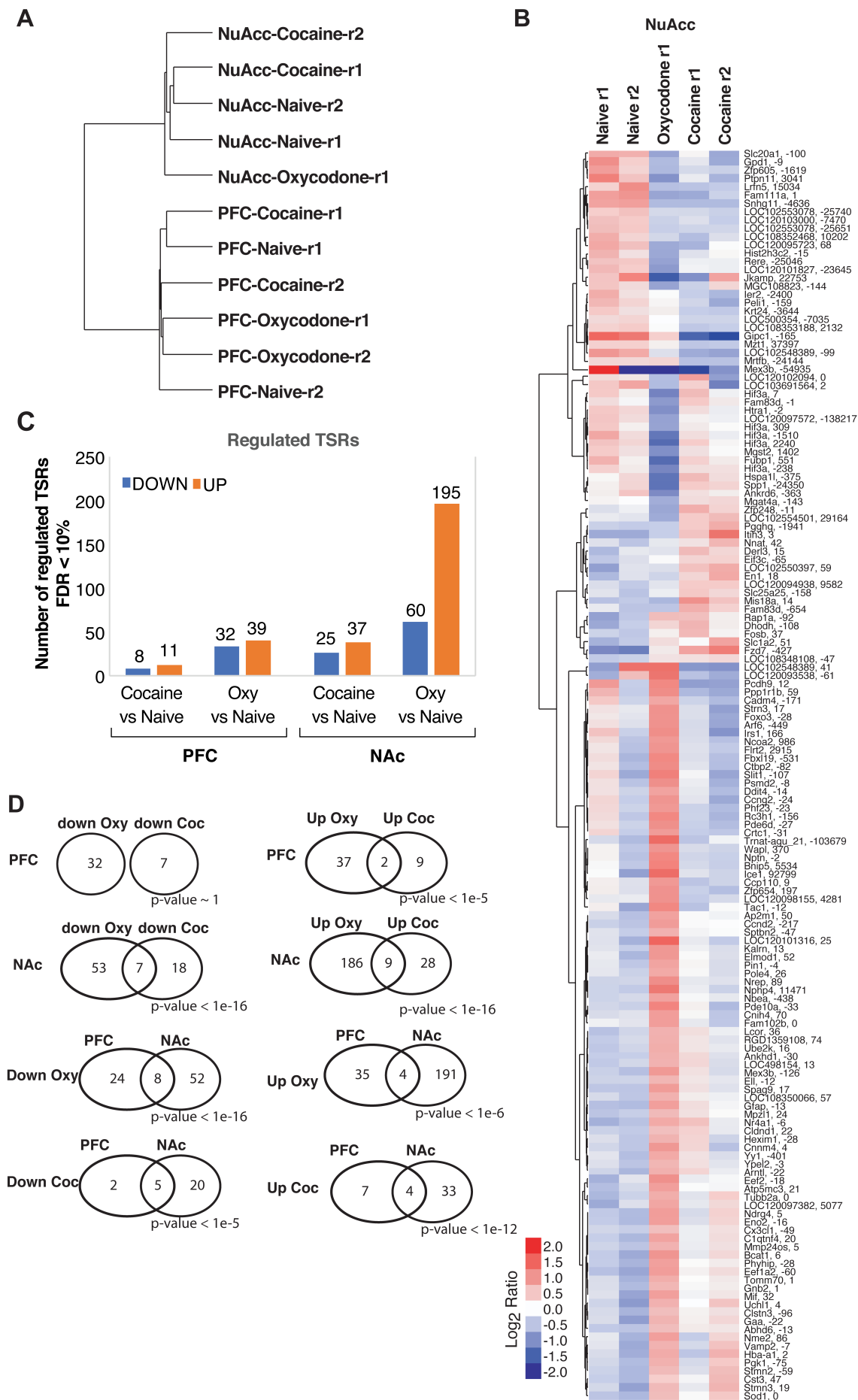

Supplement: Supplementary Figure 3 — Differentially regulated Transcriptional Start Sites (TSRs) in naïve versus cocaine- or oxycodone- exposed rat brains. (A) Hierarchical clustering of csRNA-seq samples shows segregation based on brain regions. (B) Heatmap of differential TSRs in NAc naïve, oxycodone- and cocaine-exposed rats based on log2 ratios relative to the mean; each row showing the closest gene, TSR position relative to TSS, and chromosomal coordinates. (C) Number of differentially regulated TSRs (>2 fold, FDR < 10%) across naïve and drug-exposed conditions. (D) Venn diagram showing the number of overlapping TSRs between different brain regions and drug treatment conditions. P-values for the Fisher Exact Test are reported for each Venn diagram. [file Image_3.pdf]

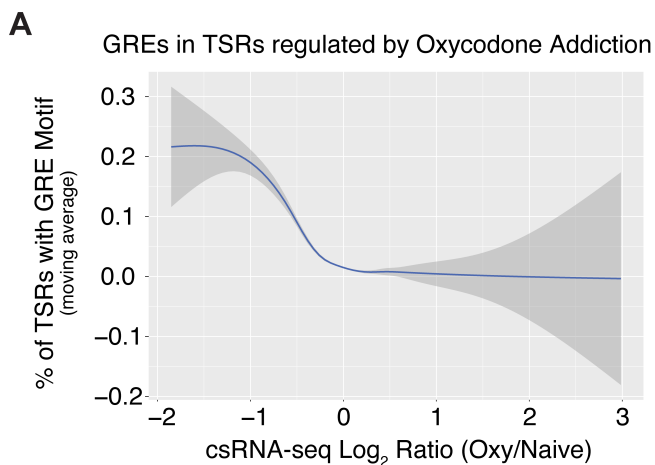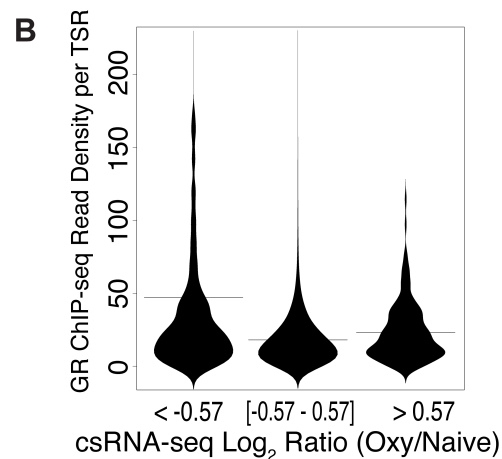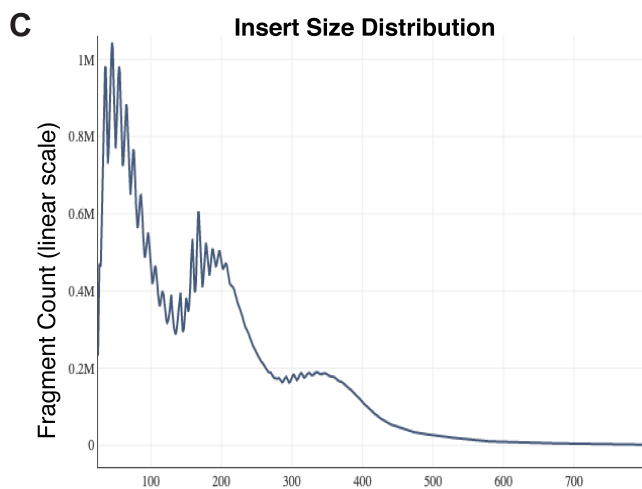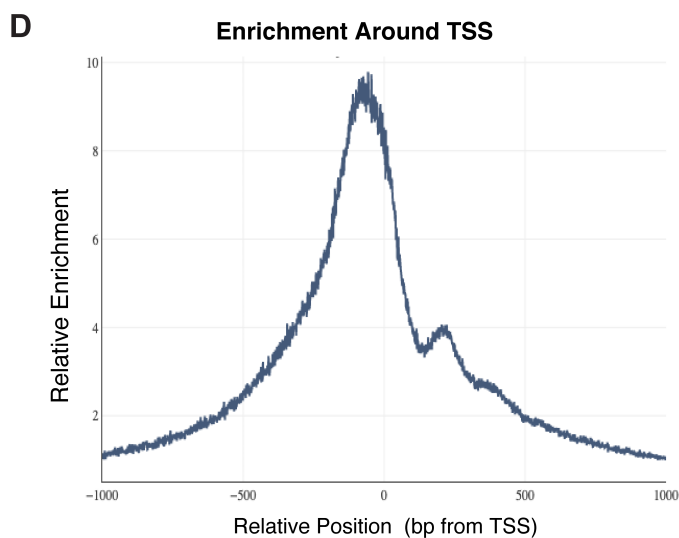

**E**

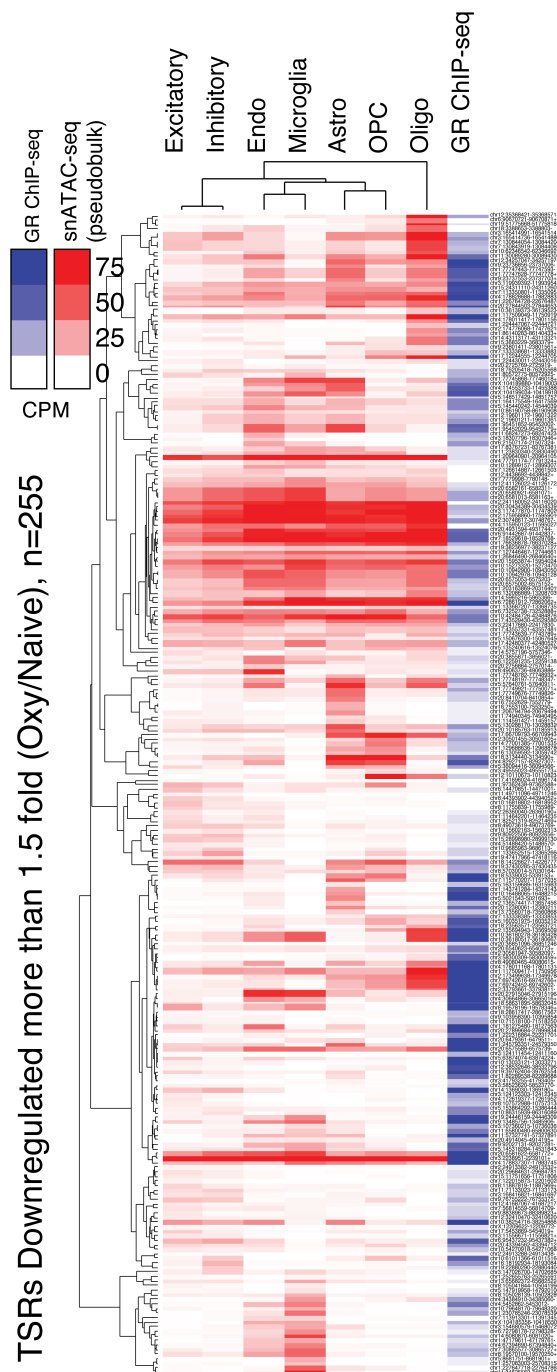

Supplement: Supplementary Figure 4 — Relationship of GR enhancers with cell type-specific open chromatin regions: (A) Fraction of TSR containing the GRE motif in TSRs regulated by oxycodone showing that ∼20% of downregulated TSRs are enriched in GRE motifs. (B) GR binding based on ChIP-seq is enriched in downregulated TSRs in oxycodone-treated vs control rats. (C) Insert size distribution of transposase accessible fragments sequenced showing expected periodicity (∼150bp). (D) Large TSS enrichment score of ∼10 is computed from the transposable sites per base in a window of 2,000 bases around annotated TSSs and shows the expected high accessibility of TSSs compared to flanking regions. [file Image_4.pdf]
